# HLola: a Very Functional Tool for Extensible Stream Runtime Verification

**DOI:** 10.1007/978-3-030-72013-1_18

**Published:** 2021-02-26

**Authors:** Felipe Gorostiaga, César Sánchez

**Affiliations:** 8 Eindhoven University of Technology, Eindhoven, The Netherlands; 9 Aalborg University, Aalborg East, Denmark; 10 IMDEA Software Institute, Madrid, Spain; 11 Universidad Politécnica de Madrid, Madrid, Spain; 12 CIFASIS, Rosario, Argentina

## Abstract

We present HLola, an extensible Stream Runtime Verification (SRV) tool, that borrows from the functional language Haskell (1) rich types for data in events and verdicts; and (2) functional features for parametrization, libraries, high-order specification transformations, etc.

SRV is a formal dynamic analysis technique that generalizes Runtime Verification (RV) algorithms from temporal logics like LTL to stream monitoring, allowing the computation of verdicts richer than Booleans (quantitative values and beyond). The keystone of SRV is the clean separation between temporal dependencies and data computations. However, in spite of this theoretical separation previous engines include hardwired implementations of just a few datatypes, requiring complex changes in the tool chain to incorporate new data types. Additionally, when previous tools implement features like parametrization these are implemented in an ad-hoc way. In contrast, HLola is implemented as a Haskell embedded DSL, borrowing datatypes and functional aspects from Haskell, resulting in an extensible engine (The tool is available open-source at http://github.com/imdea-software/hlola). We illustrate HLola through several examples, including a UAV monitoring infrastructure with predictive characteristics that has been validated in online runtime verification in real mission planning.

## 1 Introduction

Runtime Verification [4, 14, 18] is a dynamic technique that studies (1) how to generate monitors from formal specifications, and (2) algorithms to monitor the system under analysis, one trace at a time. Early RV specification languages were based on logics like past LTL [19] adapted to finite traces [5, 10, 15], regular expressions [23], fix-point logics [1], rule based languages [3], or rewriting [21]. Verdicts and many times observations in most of these specification logics are restricted to Booleans, often because most early logics in RV were borrowed from static verification—where decidability is crucial. SRV [9, 22] attempts to generalize these monitoring algorithms to richer datatypes, including in observations and verdicts. SRV offers declarative specifications where offset expressions allow accessing streams at different moments in time, including future instants. Most previous SRV developments [9, 11] and their extensions to event-based

* This work was funded in part by the Madrid Regional Government under project "S2018/TCS-4339 (BLOQUES-CM)", by Spanish National Project "BOSCO (PGC2018-102210-B-100)".
[4] The tool is available open-source at http://github.com/imdea-software/hlola systems [8,11,12,17] focus on efficiently implementing the temporal engine, promising that new datatypes can be incorporated easily. However, in practice, adding a datatype requires modifying the parser, the internal representation and the runtime system. Consequently, existing tools only support a limited hardwired collection of datatypes (typically Booleans and numeric types for quantitative monitoring).

In this paper we demonstrate the tool HLola, whose core language is Lola [9], but that enables arbitrary datatypes. HLola is implemented as an embedded DSL in Haskell. Other RV tools implemented as eDSLs include [2, 13] (in Scala), and [24] which implements LTL as an eDSL in Haskell. The main theoretical novelty of HLola is a technique called *lift deep embedding*, that consists in borrowing types transparently from Haskell and embedding the resulting language back into Haskell (see [7] for an introduction to HLola with details of the theoretical underpinnings). In fact, most HLola datatypes were introduced after the temporal engine was completed without requiring any re-implementation. An eDSL enables higher-order functions to describe transformations that produce stream declarations from stream declarations, enabling stream parametrization for free. HLola libraries collect these transformers so new logics like LTL, MTL, etc with Boolean and quantitative semantics can be implemented in a few lines (see Section 2). Haskell type-classes enable *simplifiers*, which can anticipate the value of an expression without requiring the computation of all its sub-expressions. Implementing these in previous systems requires to re-invent and implement features manually (like macro expansions, etc). HLola even allows specifications as data to implement "specifications within specifications" (a feature that allows computing a full auxiliary specification at every instant, useful in simulation and for nested properties). This is used in an UAV scenario to implement Kalman filters [16] as monitors that predict the trajectory of the unmanned aircraft. The output of this monitor is used to anticipate problems (using another monitor) and take preventive planning actions.

**Stream Runtime Verification in a nutshell** SRV generalizes monitoring algorithms to arbitrary data, where datatypes are abstracted using multi-sorted first-order interpreted signatures (called data theories in the Lola terminology). The signatures are interpreted in the sense that every functional symbol `f` used to build terms of a given type is accompanied with an evaluation function $f$ (the interpretation) that allows the computation of values (given values of the arguments). A Lola specification $\langle I, O, E\rangle$ consists of (1) a set of typed input stream variables $I$, which correspond to the inputs observed by the monitor; (2) a set of typed output stream variables $O$ which represent the outputs of the monitor as well as intermediate observations; and (3) defining equations, which associate every output $y \in O$ with a stream expression $E_y$ that describes declaratively the intended values of $y$. The set of *stream expressions* of a given type is built from constants and function symbols as constructors (as usual), and also from *offset expressions* of the form $s[k, d]$ where $s$ is a stream variable, $k$ is an integer number and $d$ is a value of the type of $s$ used as default. For example, `altitude[-1,0.0m]` represents the value of stream `altitude` in the previous step of time, with `0.0m` as default value to be used at the initial instant. Online efficient algorithms can be synthesized for specifications with (bounded) future accesses [9, 22], where efficiency means that resources (time and space) are independent of the length of the trace and can be calculated statically. HLola can be efficiently monitored in a trace-length independent sense [7].

## 2 The HLola Tool

Fig. 1 shows the software architecture of HLola. We start from an HLola specification, which can borrow datatypes, notation and features from the Haskell language (represented by the red dashed arrow in Fig. 1). A simple translator processes the specification and generates code in the Haskell eDSL. The translator does not fully parse the spec and only preforms simple rewrites, leaving most of the specification unchanged. The resulting code is combined with the HLola engine (developed in Haskell) and compiled into a binary in the target platform. A well-known downside of this approach is that during the second compilation stage, error reports may be rather cryptic. On the other hand, a Haskell expert can write specifications directly in the embedded DSL, which still resembles Lola, to finely tune an HLola specification.

The enhanced capabilities of HLola with respect to Lola (streams as data, stream type polymorphism and parametric streams) impact the syntax of the language, which diverges slightly from the syntax of the original Lola. HLola files can either be libraries or specifications: *Libraries* include HLola code that define streams and facilities to create streams, and must be declared using `library <Name>` (where `<Name>` is the name of the library) on the first line of the HLola file. *Specifications* first state the format for input and output events as `format JSON` or `format CSV`. Source files then can import libraries and stream data manipulation facilities (called theories) with the statements `use library <Name>` and `use theory <Name>` respectively. HLola files can also import arbitrary Haskell libraries using the statement `use haskell <Name>`, and include Haskell code directly anywhere within the blocks delimited between `#HASKELL` and `#ENDOFHASKELL`. Specifications then define the input and output streams. An *Input stream* is declared by its type and name in a line of the form `input <Type> <name>`, just like in the original Lola language. The syntax of `<Type>` follows the Haskell notation. An *Output stream* is specified by its type, name and parameters on the left hand side of `=`, and its defining expression on the right hand side of `=`:

```
output <TypeConstraints>? <Type> <name> <args>* = <Expr>,
```

where `<TypeConstraints>` is an optional set of constraints over the polymorphic types handled by the stream (expressed in Haskell notation), and `<args>` is an optional list of arguments of the form `<Type> <name>`. We can use `define` instead of `output` to define intermediate streams, whose values are not reported by the monitor but can be used by other streams. The defining `<Expr>` of an output stream allows the use of

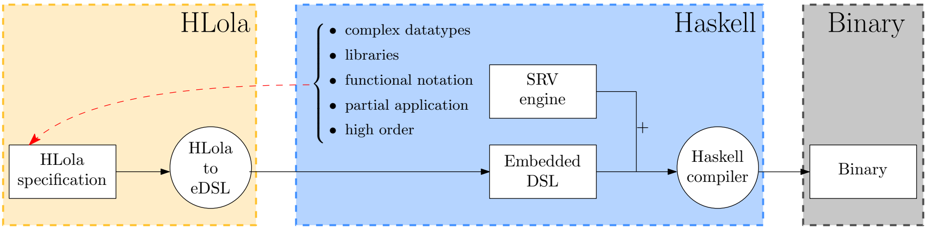

**Fig. 1.** Software Architecture of HLola.

`let` clauses, `where` blocks, type annotation, `do` notation, etc. The access to the *value* of a stream $s$ at the current instant uses the term $s$`[now]` to distinguish it from `s`, the stream itself (whose type is *stream of values*). The offset expression that accesses a stream $s$ at a shift of $i$ with default value $d$ is written as $s$`[`$i$`|`$d$`]`, as in classic Lola. The symbol `'` is used to lift an object $o$ from the theory as in `'`$o$. We sometimes indicate the arity of the object $o$ being lifted for clarity or to aid the type inference as in `2'`$o$. To improve readability, some operators have been overridden by their lifted version, such as `if-then-else`.

*Libraries.* The following HLola file defines a library of Past-LTL operators, called **LTL**, as part of the HLola distribution[5].

```
library LTL
use library Utils
output Bool historically <Stream Bool p> = p[now] && historically p [-1|'True]
output Bool once <Stream Bool p> = p[now] || once p[-1|'False]
output Bool since <Stream Bool p> <Stream Bool q> = q[now] ||
                                     (p[now] && p `since` q [-1|'False])
output Int nFalses <Stream Bool p> = nFalses p[-1|0] + if p[now] then 0 else 1
output Double percFalses <Stream Bool p> = nFalses p[now] `intdiv` (instantN[now])
```

The auxiliary library **Utils** includes `instantN`, which stores the current instant number. Stream **historically** is parametrized by **Bool**ean stream **p**. Once instantiated, `historically p` will be $true$ until `p` becomes $false$ for the first time, and will be $false$ thereafter. This definition uses offsets to define the unrolling, using the constant value $true$ in the first instant, lifted from Haskell as `'True`. This library also contains quantitative operators like **nFalses**, that counts the total number of falsifications up to an instant, and **percFalses** that calculates the ratio of falsifications. A similar library for MTL includes the parametrized definition of $\varphi\,\mathcal{U}_{(a,b)}\psi$:

```
output Bool until <(Int,Int) (a,b)> <Stream Bool phi> <Stream Bool psi> = from a
  where from a | a == b    = psi[a|'False]
               | otherwise = psi[a|'False] || (phi[a|'True] && from (a+1))
```

Here the parametrized stream **until** takes the interval $(a, b)$ and the streams $\varphi$ and $\psi$ as parameters. Similarly, the library for Quantitative MTL introduces a parametrized stream to calculate the arithmetic mean of the last $k$ values of a given stream:

```
output Double meanLast <Int k> <Stream Double str> = numr / denom
  where denom=1'fromIntegral (2'min 'k (instantN[now])) ; numr=sumLast k str [now]
```

which takes as parameters the window size **k** and the stream **str**. The denominator is the minimum of `k` and `instantN`, converted to **Double**. The numerator is the sum of the last `k` values in `str`. Polymorphosim allows us to generalize this definition to any Haskell type as long as it is Fractional, Equalizable and Streamable, using the following stream signature instead (and the same expression):

```
output (Eq a, Fractional a, Streamable a) => a meanLast <Int k> <Stream a str>
```

[5] All libraries, definitions and examples are available open-source in the GitHub repository and at https://software.imdea.org/hlola/specs.html.

## 3 Example Specifications

In this section we show a collection of HLola specifications to demonstrate the capabilities of HLola to define stream based monitors.

*Temporal Logics.* HLola allows us to easily define, in a declarative way, many specifications written in temporal logic. The HLola distribution contains many LTL examples, including a sender/receiver model from [6], and other temporal logics. Consider the following MTL property from [20]: $\square(alarm \rightarrow (\diamondsuit_{[0,10]} allClear \vee \diamondsuit_{[10,10]} shutdown))$, which includes deadlines between environment events and the corresponding system responses, stating that that an *alarm* is followed by a *shutdown* event in exactly 10 time units unless *allClear* is received. This is defined in HLola as follows:

```
format JSON
use library MTL
#HASKELL
data Event = Alarm | AllClear | ShutDown deriving (Generic,Read,FromJSON,Eq)
#ENDOFHASKELL
input Event event
define Bool allClear = event [now] === 'AllClear
define Bool shutdown = event [now] === 'Shutdown
define Bool alarm    = event [now] === 'Alarm
output Bool property = alarm [now] `implies` (willClear[now] || willShutdown[now])
  where willClear    = eventually (0,10) allClear
        willShutdown = eventually (10,10) shutdown
```

*Pinescript example.* TradingView is an online charting platform for stock exchange, which offers the Pinescript language to query stock time series. Pinescript queries are then run in the company's servers. We have implemented the indicators of Pinescript in HLola as a library, and we have implementated a trading strategy[6] using the HLola Pinescript library. Compared to Pinescript, HLola offers formal semantics, runtime resource guarantees (time and space) and is much more expressive, for example allowing relational queries that involve multiple stocks (their averages, etc).

*UAV specifications.* We have used HLola also for the online monitoring of several properties of UAVs missions. For example: (1) That the UAV does not fly over forbidden regions, and (2) that the UAV is in good position when it takes a picture. The input streams of these two specifications consist of the state of the UAV at every instant and the onboard camera events to detect when a picture is being captured. This specification imports geometric facilities from `theory Geometry2D`, and Haskell libraries `Data.Maybe` and `Data.List`. It then defines custom datatypes to retrieve data from the UAV, which are enclosed in a verbatim `HASKELL` block. The output stream `all_ok_capturing` assesses that, whenever the vehicle is taking a picture, the height, roll and pitch are acceptable and the vehicle is near the target location. The output stream `flying_in_safe_zones` reports if the UAV is flying outside every forbidden region. The output stream `depth_into_poly` takes the minimum of the distances between the vehicle position and every side of the forbidden region inside which the vehicle is.

[6] Available at www.tradingview.com/script/DushajXt-MACD-Strategy

```
format JSON
use theory Geometry2D
use library Utils
use haskell Data.Maybe
use haskell Data.List
#HASKELL
data Attitude = Attitude {yaw :: Double, roll :: Double, pitch :: Double}
                         deriving (Show,Generic,Read,FromJSON,ToJSON)
data Target   = Target {x :: Double, y :: Double, num_wp :: Double} ...
data Position = Position {x :: Double, y :: Double, alt :: Double} ...
#ENDOFHASKELL
input     Attitude attitude
input      Vector2 velocity
input     Position position
input       Double altitude
input       Target target
input [[[Double]]] nofly
input     [String] events_within output Bool all_ok_capturing = capturing [now] `implies`
  (height_ok [now] && near [now] && roll_ok [now] && pitch_ok [now])
output Bool flying_in_safe_zones = 'isNothing (flying_in_poly [now])
output (Maybe Double) depth_into_poly = let
  mSides = '(fmap polygonSides) (flying_in_poly [now])
  distance_from_pos = 'shortestDist (filtered_pos [now])
  in    2'fmap distance_from_pos mSides
  where shortestDist x = minimum.map (distancePointSegment x)
define Bool capturing = ...
define Double filtered_pos_component <(Position->Double) field> <String nm> = ...
define Double filtered_pos_x   = filtered_pos_component x "x" [now]
define Double filtered_pos_y   = filtered_pos_component y "y" [now]
define Double filtered_pos_alt = filtered_pos_component alt "alt" [now]
define Point2 filtered_pos = 'P (filtered_pos_x [now]) (filtered_pos_y [now])
define Bool near = let target_pos = 'targetToPoint (target [now])
  in    2'distance (filtered_pos [now]) target_pos < 1
  where targetToPoint (Target x y _) = P x y
define Bool height_ok = filtered_pos_alt [now] > 0
define Bool roll_ok   = '(abs.roll) (attitude [now]) < 0.0523
define Bool pitch_ok  = '(abs.pitch) (attitude [now]) < 0.0523
define [Polygon] no_fly_polys = ...
define (Maybe Polygon) flying_in_poly = let
  position_in_poly = 'pointInPoly (filtered_pos [now])
  in 2'find position_in_poly (no_fly_polys [now])
```

Intermediate stream **capturing** captures whether the UAV is taking a picture (omitted for brevity). The streams **filtered_pos_alt** and **filtered_pos** represent the location and altitude of the UAV filtered to reduce noise from the sensors. We omit the definition of the filter, which is implemented in **filtered_pos_component** The streams **height_ok**, **roll_ok**, and **pitch_ok**, calculate that the corresponding attitude of the vehicle is within certain boundaries. Finally, the intermediate stream **no_fly_polys** obtains a set of Polygons from the input forbidden regions (its definition has been omitted), and the stream **flying_in_poly** returns the forbidden region in which the vehicle is flying, if any. The artifact attached to this paper includes more UAV specifications, which have been validated in real missions [25].

## References

[1] Howard Barringer, Allen Goldberg, Klaus Havelund, and Koushik Sen. Rule-based runtime verification. In *Proc. of the 5th Int’l Conf. on Verification, Model Checking and Abstract Interpretation (VMCAI’04)*, volume 2937 of *LNCS*, pages 44–57. Springer, 2004.

[2] Howard Barringer and Klaus Havelund. Tracecontract: A scala DSL for trace analysis. In Michael J. Butler and Wolfram Schulte, editors, *FM 2011: Formal Methods - 17th International Symposium on Formal Methods, Limerick, Ireland, June 20-24, 2011. Proceedings*, volume 6664 of *Lecture Notes in Computer Science*, pages 57–72. Springer, 2011.

[3] Howard Barringer, David Rydeheard, and Klaus Havelund. Rule systems for run-time monitoring: From eagle to ruler. In Oleg Sokolsky and Serdar Taşıran, editors, *Runtime Verification*, pages 111–125, Berlin, Heidelberg, 2007. Springer Berlin Heidelberg.

[4] Ezio Bartocci and Yliès Falcone, editors. *Lectures on Runtime Verification - Introductory and Advanced Topics*, volume 10457 of *LNCS*. Springer, 2018.

[5] Andreas Bauer, Martin Leucker, and Chrisitan Schallhart. Runtime verification for LTL and TLTL. *ACM Transactions on Software Engineering and Methodology*, 20(4):14, 2011.

[6] Marco Benedetti and Alessandro Cimatti. Bounded model checking for past LTL. In *Proc. of TACAS’03*, volume 2619 of *LNCS*, pages 18–33. Springer, 2003.

[7] Martín Ceresa, Felipe Gorostiaga, and César Sánchez. Declarative stream runtime verification (hlola). In Bruno C. d. S. Oliveira, editor, *Programming Languages and Systems*, pages 25–43, Cham, 2020. Springer International Publishing.

[8] Lukas Convent, Sebastian Hungerecker, Martin Leucker, Torben Scheffel, Malte Schmitz, and Daniel Thoma. TeSSLa: Temporal stream-based specification language. In *Proc. of SBMF’18*, volume 11254 of *LNCS*. Springer, 2018.

[9] Ben D’Angelo, Sriram Sankaranarayanan, César Sánchez, Will Robinson, Bernd Finkbeiner, Henny B. Sipma, Sandeep Mehrotra, and Zohar Manna. LOLA: Runtime monitoring of synchronous systems. In *Proc. of the 12th Int’l Symp. of Temporal Representation and Reasoning (TIME’05)*, pages 166–174. IEEE CS Press, 2005.

[10] Cindy Eisner, Dana Fisman, John Havlicek, Yoad Lustig, Anthony McIsaac, and David Van Campenhout. Reasoning with temporal logic on truncated paths. In *Proc. of the 15th Int’l Conf. on Computer Aided Verification (CAV’03)*, volume 2725 of *LNCS*, pages 27–39. Springer, 2003.

[11] Peter Faymonville, Bernd Finkbeiner, Malte Schledjewski, Maximilian Schwenger, Marvin Stenger, Leander Tentrup, and Torfah Hazem. StreamLAB: Stream-based monitoring of cyber-physical systems. In *Proc. of the 31st Int’l Conf. on Computer-Aided Verification (CAV’19)*, volume 11561 of *LNCS*, pages 421–431. Springer, 2019.

[12] Felipe Gorostiaga and César Sánchez. Striver: Stream runtime verification for real-time event-streams. In *Proc. of the 18th Int’l Conf. on Runtime Verification (RV’18)*, volume 11237 of *LNCS*, pages 282–298. Springer, 2018.

[13] Klaus Havelund. Rule-based runtime verification revisited. *Int. J. Softw. Tools Technol. Transf.*, 17(2):143–170, 2015.

[14] Klaus Havelund and Allen Goldberg. Verify your runs. In *Proc. of VSTTE’05*, LNCS 4171, pages 374–383. Springer, 2005.

[15] Klaus Havelund and Grigore Roşu. Synthesizing monitors for safety properties. In *Proc. of the 8th Int’l Conf. on Tools and Algorithms for the Construction and Analysis of Systems (TACAS’02)*, volume 2280 of *LNCS*, pages 342–356. Springer-Verlag, 2002.

[16] Rudolph Emil Kalman. A new approach to linear filtering and prediction problems. *Transactions of the ASME–Journal of Basic Engineering*, 82(Series D):35–45, 1960.

[17] Martin Leucker, César Sánchez, Torben Scheffel, Malte Schmitz, and Alexander Schramm. TeSSLa: Runtime verification of non-synchronized real-time streams. In *Proc. of the 33rd Symposium on Applied Computing (SAC’18)*. ACM, 2018.

[18] Martin Leucker and Christian Schallhart. A brief account of runtime verification. *J. Logic Algebr. Progr.*, 78(5):293–303, 2009.

[19] Zohar Manna and Amir Pnueli. *Temporal Verification of Reactive Systems*. Springer-Verlag, 1995.

[20] Joël Ouaknine and James Worrell. Some recent results in metric temporal logic. In *Proc. of FORMATS’08*, volume 5215 of *LNCS*, pages 1–13. Springer, 2008.

[21] Grigore Roşu and Klaus Havelund. Rewriting-based techniques for runtime verification. *Automated Software Engineering*, 12(2):151–197, 2005.

[22] César Sánchez. Online and offline stream runtime verification of synchronous systems. In *Proc. of the 18th Int’l Conf. on Runtime Verification (RV’18)*, volume 11237 of *LNCS*, pages 138–163. Springer, 2018.

[23] Koushik Sen and Grigore Roşu. Generating optimal monitors for extended regular expressions. In Oleg Sokolsky and Mahesh Viswanathan, editors, *Electronic Notes in Theoretical Computer Science*, volume 89. Elsevier, 2003.

[24] Volker Stolz and Frank Huch. Runtime verification of concurrent haskell programs. *Electron. Notes Theor. Comput. Sci.*, 113:201–216, 2005.

[25] Sebastián Zudaire, Felipe Gorostiaga, César Sánchez, Gerardo Schneider, and Sebastián Uchitel. Assumption monitoring using runtime verification for UAV temporal task plan executions. Under submission, 2020.

